# Evaluation of the relationship between GPR43 and adiposity in human

**DOI:** 10.1186/1743-7075-10-11

**Published:** 2013-01-17

**Authors:** Evelyne M Dewulf, Qian Ge, Laure B Bindels, Florence M Sohet, Patrice D Cani, Sonia M Brichard, Nathalie M Delzenne

**Affiliations:** 1 Metabolism and Nutrition Research Group, LDRI, Université catholique de Louvain, Avenue E. Mounier 73, PO box B1.73.11, 1200, Brussels, Belgium; 2 Endocrinology and Metabolism Unit, IREC, Université catholique de Louvain, Brussels, Belgium

**Keywords:** Human, GPR43, Adipocyte differentiation, Obesity, Inflammation

## Abstract

**Background:**

GPR43 is a G-protein-coupled receptor that participates in adipocyte differentiation in mice and is over-expressed in adipose tissue of obese mice. The aim of this study was to investigate the implication of GPR43 in adipogenesis in humans and to determine the influence of obesity on its expression in human adipose tissue.

**Findings:**

Preadipocytes were isolated from human omental adipose tissue and cultured during 13 days. One PPARγ agonist (troglitazone) and three GPR43 agonists (two physiological and one synthetic) were tested for their ability to induce differentiation. After 13 days, the three GPR43 agonists had no impact on aP2 expression, a marker of adipocyte differentiation, whereas troglitazone led to a huge over-expression of aP2 in these cells but tended to decrease GPR43 expression (p=0.06).

GPR43 and inflammatory markers expression was also quantified in omental adipose tissue from lean and obese individuals. GPR43 expression in total adipose tissue was similar between obese patients and lean subjects and did not correlate with aP2 expression. In contrast, GPR43 expression positively correlated with TNFα mRNA.

**Conclusions:**

Our results suggest the absence of relationship between GPR43 and adipocyte differentiation in humans, unlike what was observed in mice. Furthermore, GPR43 expression is not increased in adipose tissue from obese subjects but could be related to TNFα-related inflammatory processes.

## Findings

### Introduction

GPR43, also known as free fatty acid receptor 2 (FFA2), is a G-protein-coupled receptor activated by short-chain fatty acids, i.e. acetate and propionate, produced through fermentation of non-digestible carbohydrates [1-3]. It is highly expressed in immune cells but is also present in other tissues (e.g. adipose tissue, spleen, bone marrow, intestine, liver) [4] and seems implicated in adipose tissue metabolism. GPR43 activation by acetate and propionate *in vitro* stimulates adipocyte differentiation in 3T3-L1 preadipocytes. 3T3-L1 cells transfected with GPR43 siRNA exhibit decreased expression of peroxisome proliferator-activated receptor gamma (PPARγ) -the master regulator of adipogenesis- and less fat accumulation [5]. Moreover, acetate and propionate inhibit *in vitro* and *in vivo* lipolysis by activating GPR43 [5,6]. In mice fed a high-fat diet, the augmented adiposity and adipocyte enlargement have been associated with an over-expression of PPARγ target genes and GPR43 in subcutaneous adipose tissue. Furthermore, a PPARγ agonist increased GPR43 expression in explants of mouse subcutaneous adipose tissue thus suggesting PPARγ as a driver of GPR43 expression [7]. Altogether, these results suggest a potential link between GPR43 expression and adipocyte differentiation in mice. However, in humans, the implication of this receptor in adipogenesis and its expression in obesity has not been studied yet.

### Methods and materials

#### Chemicals

All chemicals were purchased from Sigma-Aldrich (Saint Louis, MO, USA), except the synthetic GPR43 agonist [4-chloro-α-(1-methyl ethyl)-*N*-2-thiazolyl-benzeneacetamide] (CMTB) [8] (Ambinter, Paris, France) and collagenase A (Roche Diagnostics Belgium, Vilvoorde, Belgium).

#### Isolation and culture of stromal-vascular cells from human adipose tissue

Omental adipose tissue (OAT) from 4 obese patients undergoing abdominal surgery were fractionated into adipocytes and stromal-vascular cells (SVC) as previously described [9,10]. Briefly, fat tissue was cut into small pieces and incubated for 15 min in a shaking water bath at 37°C in KREBS-BSA 2% with collagenase A (8.33 mg/g tissue). Digested tissue was filtered and centrifuged at 400 g for 1 min. The infranatant containing the SVC was washed three times. Preadipocytes were grown to confluence in DMEM-F-12 medium (Invitrogen, Life Technologies, Gent, Belgium) with 10% fetal bovine serum (PAA Laboratories, Pasching, Austria), streptomycin 100 μg/ml and penicillin 100 IU/ml (Gibco, Inchinnan, Scotland) at 37°C in humidified 5% CO_2_ and then differentiated *in vitro* during 13 days using a chemically defined serum-free medium consisting of DMEM-F-12 (1:1) supplemented with 15 mM HEPES, 15 mM NaHC0_3_, 33 μM biotin, 17 μM panthotenate, 10 μg/ml apotransferrin, 66 nM insulin, 1 μM dexamethasone, 200 pM triiodothyronine, and antibiotics. Isobutylmethylxanthine (500 μM) was added during the first 3 days to induce differentiation. During differentiation, different conditions were tested: control medium (CT), troglitazone (TZD) (10 μM), acetate (10 μM), propionate (10 μM) and CMTB (1 μM). DMSO was used to dilute TZD and CMTB and added to each medium to obtain the same final concentration (0.13%). Cells were then harvested and frozen at −80°C until subsequent mRNA analysis.

#### Isolation of human adipose tissue

OAT was obtained from 10 lean and 23 obese patients undergoing abdominal surgery after an overnight fast, as described elsewhere [10]. Descriptive characteristics of these patients are given in Table 1.

**Table 1:** Descriptive characteristics of lean and obese patients

	**Men/Women**	**Age (years)**	**BMI (kg/m**^**2**^**)**
Lean	4/6	60 ± 4	23.1 ± 0.4
Obese	12/11	42 ± 3	45.1 ± 1.5

Data are mean ± SEM. BMI: body mass index.

#### Real-time quantitative PCR

Total RNA was isolated using a TriPure Isolation Reagent Kit (Roche Diagnostics Belgium, Vilvoorde, Belgium), cDNA was prepared by reverse transcription of 100 ng total RNA using a Reverse Transcription System Kit (Promega, Madison, WI, USA) and quantitative PCR was performed as previously described [7]. Primers used to detect the targeted genes are available upon request.

#### Statistical analysis

Results are presented as mean ± SEM. Statistical significance of difference was assessed by one-way ANOVA followed by post hoc Tukey’s multiple comparison test when comparing 3 groups or more, or by a Student *t*-test when comparing 2 groups. Correlations were analyzed using Pearson’s correlation test in GraphPad Prism (version 5.00 for Windows GraphPad Software, San Diego, CA, USA). The level of significance was set at p< 0.05.

### Results and discussion

#### Implication of human GPR43 in adipogenesis

**Figure 1:**
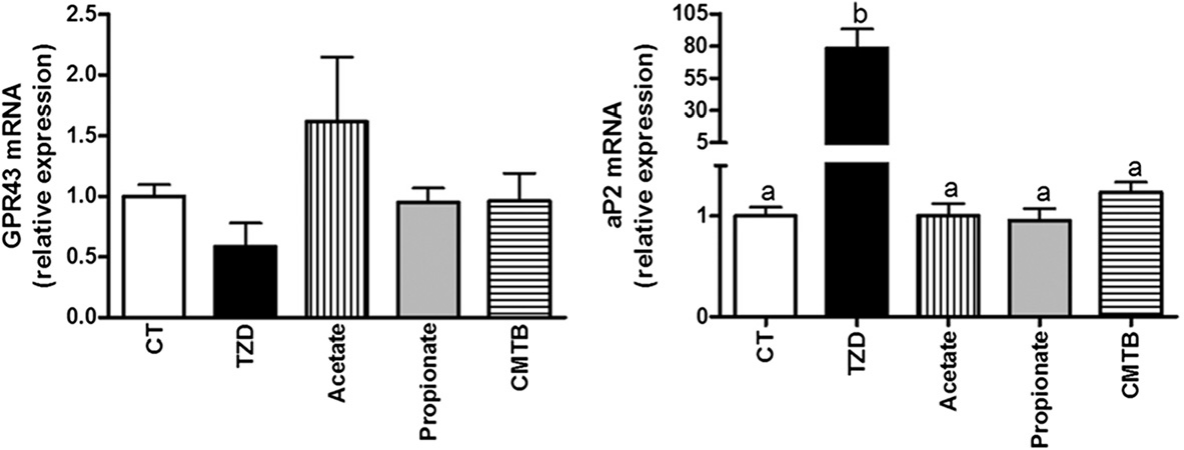
**Study of GPR43 involvement in human adipogenesis.** GPR43 and aP2 mRNA levels in human preadipocytes, 13 days after induction of differentiation in presence of the vehicle (CT) or different pharmacological agents: troglitazone (TZD), acetate, propionate and CMTB. Mean value obtained in the CT group (i.e.; medium without the addition of the pharmacological agents) is set at 1. Data are mean ± SEM for 4 different cultures. Data with different superscript letters are significantly different with p<0.001, according to one-way ANOVA followed by post hoc Tukey’s multiple comparison test. GPR43: G-protein-coupled receptor 43.

GPR43 is implicated in adipocyte differentiation in mice. Acetate and propionate, two physiological agonists of this receptor, induce adipocyte differentiation in 3T3-L1 cells [5]. *In vitro* and *ex vivo* experiments also suggest a link between PPARγ activity, a master regulator of adipogenesis, and GPR43 expression since the incubation in the presence of a PPARγ agonist induces GPR43 over-expression in 3T3-L1 adipocytes [5] and explants of mouse adipose tissue [7]. In this study, we investigated the influence of GPR43 agonists on adipocyte differentiation in humans. We used preadipocytes isolated from human OAT. These cells were cultured during 13 days with different treatments that could potentially induce differentiation: a PPARγ agonist (TZD), acetate, propionate or one synthetic GPR43 agonist, CMTB. We evaluated the influence of these treatments on the expression of GPR43 and aP2, a PPARγ target gene known as a late marker of adipocyte differentiation [11] (Figure 1). TZD induced an 80-fold increase in aP2 expression thus signing an important induction of differentiation following PPARγ activation. Surprisingly, TZD tended to decrease GPR43 expression (p=0.06). This effect was opposite to that previously demonstrated in mice [5,7]. Moreover, the three GPR43 agonists did not significantly modulate the expression of our genes of interest. These results allow us to propose that, in humans, GPR43 may not be implicated in the process of adipogenesis. Indeed, an over-expression of aP2 -reflecting an increased differentiation- is not associated with GPR43 over-expression and GPR43 agonists are not able to induce differentiation in human preadipocytes as demonstrated by the lack of effect on aP2 expression. These are preliminary results that should be confirmed, e.g. by showing that blocking GPR43 has no impact on the TZD-induced differentiation but so far, no GPR43 antagonist exists.

#### GPR43 Expression in human adipose tissue: link with inflammation

**Figure 2:**
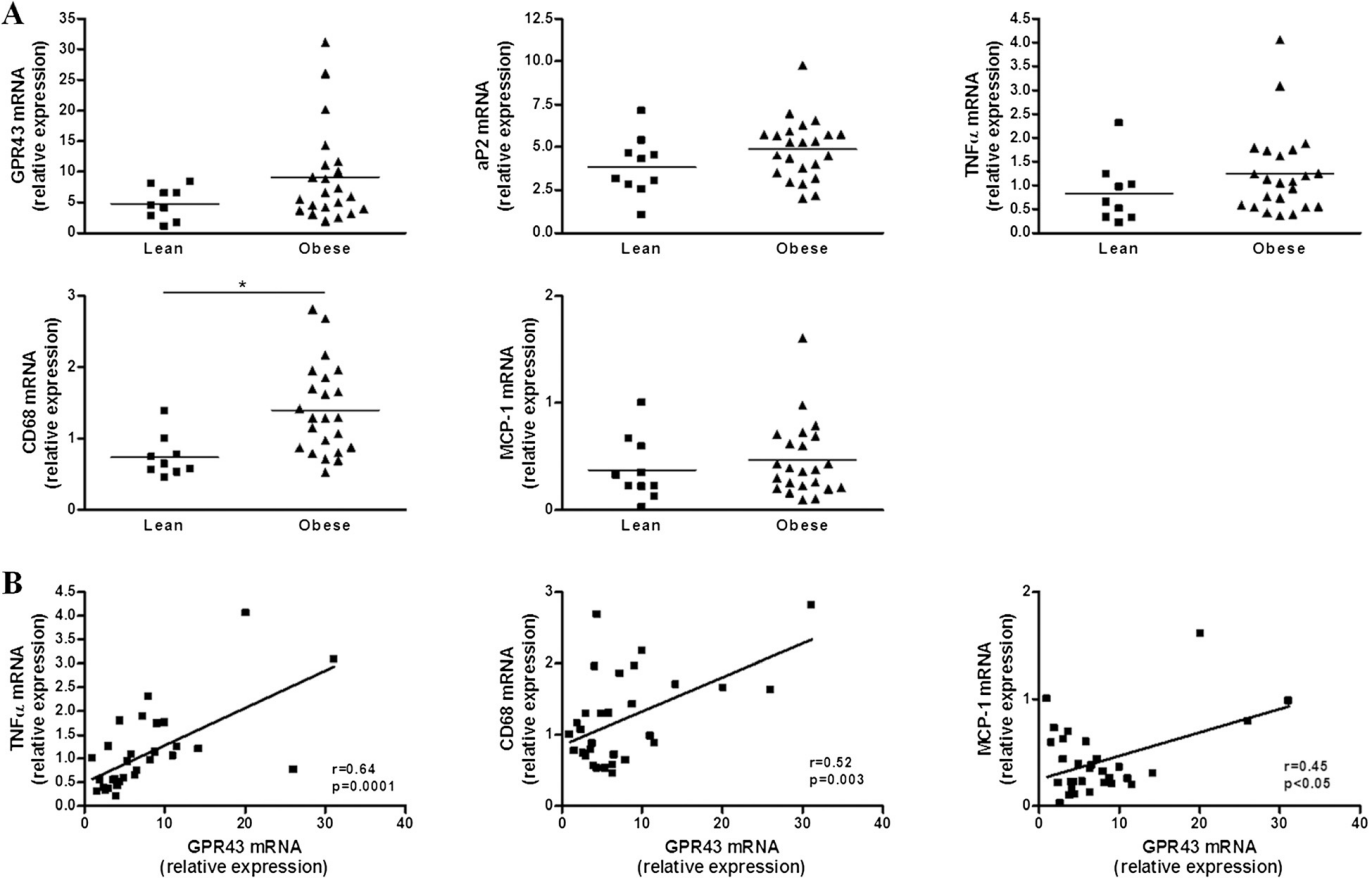
**Influence of obesity on GPR43 expression in human adipose tissue. A.** GPR43, aP2, TNFα, CD68 and MCP-1 mRNA levels in human omental adipose tissue collected from 10 lean patients and 23 obese patients. Data are mean ± SEM, * p<0.01 according to the Student’s *t*-test. **B.** Correlations between GPR43 mRNA and TNFα, CD68 and MCP-1 mRNAs, r and p values according to the Pearson’s correlation test. GPR43: G-protein-coupled receptor 43; TNFα: tumor necrosis factor α; CD68: cluster of differentiation 68; MCP-1: monocyte chemoattractant protein-1.

Hong *et al.* reported that GPR43 was over-expressed in adipose tissue of high-fat diet-fed mice [5]. We demonstrated that this over-expression was associated with an increased adipogenesis and augmented expression of inflammatory markers [7]. However, so far, GPR43 expression has never been evaluated in adipose tissue of obese humans. Therefore, in human OAT collected from 10 lean and 23 obese patients, we investigated the expression of GPR43 but also aP2, TNFα, CD68 and MCP-1 mRNAs as markers of adipocyte differentiation, inflammation, macrophages and inflammatory cells recruitment, respectively [12,13] (Figure 2A). In comparison to lean subjects, obese patients exhibited no significant increase in GPR43 expression, despite an important variability, and a significant over-expression of CD68, thus suggesting a higher macrophages content, a well-established characteristic of obese adipose tissue [14]. On the other hand, aP2, TNFα and MCP-1 mRNAs were not significantly modified in obese subjects compared to lean individuals. Interestingly, there was no correlation between GPR43 mRNA and aP2 mRNA (Pearson r = −0.10, p = 0.61) thus strengthening our hypothesis on the absence of link between these two genes in human adipose tissue. In contrast, we observed significant and positive correlations between GPR43 mRNA on one hand and CD68, TNFα and MCP-1 expression on the other hand (Figure 2B). However, it is worth noting that, when removing the 3 obese patients with the highest GPR43 expression (outliers), the sole correlation remaining significant was between GPR43 and TNFα mRNAs (Pearson r = 0.57, p = 0.003). Thus, unlike what has been observed in mice, human obesity is not associated with an over-expression of GPR43 in OAT. This receptor may not be implicated in adipogenesis but would rather be linked to inflammatory processes in adipose tissue, as suggested by the strong correlation between GPR43 and TNFα mRNAs. Interestingly, a link between GPR43 and inflammation has already been proposed in some inflammatory diseases [15]. In models of colitis, arthritis and asthma, Maslowski *et al.* highlighted an exacerbated inflammation in GPR43-deficient mice as compared to wild-type mice, thus suggesting a beneficial role of GPR43 in these inflammatory processes [16]. However, another study showed that GPR43 knockout mice were protected against chronic Dextran Sulfate Sodium-induced colitis thus proposing the opposite conclusion [17]. Therefore, the beneficial or deleterious implication of GPR43 in inflammation is still controversial and our preliminary results suggest adipose tissue as a new potential target to focus on when studying GPR43 and inflammation in humans.

### Conclusion

In this first study focusing on GPR43 in human adipose tissue, some data previously shown in mice, i.e. the implication of GPR43 in the process of adipocyte differentiation, have not been confirmed. Indeed, GPR43 does not seem implicated in human adipogenesis as its ligands do not induce differentiation of preadipocytes. Moreover, GPR43 expression is not induced by a PPARγ agonist and is not correlated to aP2 expression, a well-known marker of adipocyte differentiation. However, although GPR43 is not over-expressed in the OAT of all obese individuals, its expression seems associated with TNFα-related inflammatory process. Larger studies are needed in order to evaluate the co-regulation of GPR43 and TNFα expression in human adipose tissue.

## Abbreviations

CD68: Cluster of differentiation 68; CMTB: 4-chloro-α-(1-methyl ethyl)-*N*-2-thiazolyl-benzeneacetamide; CT: Control; GPR43: G-protein-coupled receptor 43; MCP-1: Monocyte chemoattractant protein-1; OAT: Omental adipose tissue; PPARγ: Peroxisome proliferator-activated receptor gamma; SVC: Stromal-vascular cells; TNFα: Tumor necrosis factor alpha.

## Competing interests

The authors declare no conflict of interest.

## Authors’ contributions

Design of experiments: EMD, SMB, NMD; performance of experiments: EMD, QG, LBB, FMS; data analysis and interpretation: EMD, LBB, PDC, SMB, NMD; manuscript writing: EMD, NMD. All authors have read, revised and approved the final manuscript.
